# Causal roles of immune cells and metabolites in chronic pancreatitis: a mendelian randomization study

**DOI:** 10.1186/s41065-025-00378-8

**Published:** 2025-02-12

**Authors:** Chao Zhang, Tao Yang, Yuan Yu, Qian Jia, Wan-Meng Xiao, Sha Liu, Ze-Hui Yu, Cheng-Li Wen, Yan Wei, Hao Li, Mu-Han Lü

**Affiliations:** 1 Department of Gastroenterology, The Affiliated Hospital, Southwest Medical University, Luzhou, Sichuan Province China; 2 Gulin County People’s Hospital, Luzhou, Sichuan Province China; 3 Laboratory Animal Center, Southwest Medical University, Luzhou, Sichuan China; 4 Animal and Human Disease Research of Luzhou Key Laboratory, Luzhou, China; 5 Department of Critical Care Medicine, The Affiliated Hospital, Southwest Medical University, Luzhou, China; 6 Key Laboratory of Medical Electrophysiology, Ministry of Education & Medical Electrophysiological Key Laboratory of Sichuan Province, Collaborative Innovation Center for Prevention of Cardiovascular Diseases), Institute of Cardiovascular Research, Southwest Medical University, Luzhou, Sichuan Province China

## Abstract

**Background:**

Previous research has established a correlation between immune cells and an increased likelihood of Chronic pancreatitis (CP). However, studies investigating the causal relationship remain limited.

**Methods:**

This study utilized publicly available genome-wide association study (GWAS) databases and conducted a two-sample Mendelian randomization (MR) analysis to examine the causal relationships (CRs) among 731 immune cells, 1,400 metabolites, and CP. Mediation MR analysis was also performed to assess whether metabolites serve as mediators in the relationship between immune cells and CP.

**Results:**

Our study identified four immune cell types that act as risk factors for CP, with odds ratios (OR) ranging between 1.076 and 1.177. In contrast, three immune cell types were found to serve as protective factors, exhibiting OR values between 0.846 and 0.913. Additionally, four metabolites were implicated as risk factors for CP, with OR values ranging from 1.243 to 1.334. On the other hand, eight metabolites were discovered to have a protective effect, with OR values between 0.580 and 0.871. Mediation analysis revealed that cholesterol levels mediate the causal relationship between immune cell cells and CP, with a mediation effect of 0.00918, accounting for 9.18% of the total effect.

**Conclusions:**

Our findings provide valuable insights into the genetic underpinnings of CP, highlighting the role of immune cells and plasma metabolites in its pathogenesis. The mediation analysis further suggests that the presence of CD25 on IgD-CD38-B cells may facilitate CP development through the elevation of cholesterol levels. These results not only deepen our understanding of CP but also suggest potential biological targets for therapeutic intervention. Future clinical research should focus on these mediators to develop more effective treatment strategies for CP.

**Supplementary Information:**

The online version contains supplementary material available at 10.1186/s41065-025-00378-8.

## Introduction

Chronic pancreatitis (CP) is a progressive, fibrotic inflammatory disease characterized by recurring episodes of acute pancreatitis and ongoing inflammation. Clinically, CP presents as chronic abdominal pain and the deterioration of both endocrine and exocrine pancreatic functions [1]. The annual incidence of CP is estimated to range from 4 to 14 cases per 100,000 individuals, with a prevalence of 40 to 60 cases per 100,000 individuals [2–4]. CP significantly impacts patients’ quality of life, reduces life expectancy, and is an important risk factor for pancreatic cancer [5]. Due to the absence of specific clinical features in the early stages, CP diagnosis primarily depends on identifying morphological or functional changes [6]. Therefore, recognizing risk factors and early biomarkers for CP is crucial.

As CP progresses, inflammation affects more acinar cells, which are gradually replaced by fibrotic tissue. Although several risk factors, including chronic heavy alcohol use, smoking, gallstones, and genetic factors, have been identified [7], the precise causes of CP remain unclear. In recent years, the role of immune cells in CP progression has received increasing attention [8]. Immunohistochemical studies have shown significantly higher levels of monocytes and T lymphocytes in CP tissue compared to normal pancreatic tissue, with CD8^+^ T cells being the predominant T-cell subtype [9]. Another study utilizing single-cell sequencing found notable differences in immune cell composition between CP and normal pancreatic tissue, with myeloid cells predominating in normal tissue and T cells elevated in CP tissue [10]. These findings suggest that immune cells play a vital role in CP development. However, the precise role of immune cells, particularly adaptive immune cells, in CP etiology is still not well understood.

In addition to the involvement of immune cells, metabolic factors also contribute significantly to CP progression [11]. Plasma metabolites related to triglycerides, such as free fatty acids, very low-density lipoprotein, and low-density lipoprotein, have strong associations with CP progression [12]. A large observational study revealed that when plasma triglyceride levels exceed 20 mmol/L, the risk of CP increases by 25-fold compared to normal levels [13]. Another study demonstrated that obesity, hyperlipidemia, and hyperglycemia accelerate CP progression [14]. Additionally, plasma metabolites like β-carotene, behenic acid, indole-3-acetic acid, and hippuric acid have been identified as early biomarkers for CP [15]. Although these studies suggest an association between plasma metabolites and CP, most are retrospective or observational, and do not clarify whether a causal relationship (CR) exists.

Mendelian randomization (MR), which utilizes genetic variation, provides a robust approach to examining causal relationships (CRs) between exposures and outcomes, effectively reducing confound and avoiding reverse causation [16]. Compared to randomized controlled trials, MR studies are generally more feasible and cost-effective [17]. In this study, we employed a two-step MR approach to investigate potential CRs between immune cells and CP, along with the mediating role of plasma metabolites. First, we examined the CR between 731 immune cell types and CP. Next, we assessed the CR between 1,400 plasma metabolites and CP. Finally, we investigated the potential mediating role of plasma metabolites in the relationship between immune cells and chronic pancreatitis. This research is the first to highlight the involvement of adaptive immune cells, specifically CD8^+^ T cells and B cells, in the progression of CP, while also identifying metabolites as key intermediaries. Our findings deepen the understanding of chronic pancreatitis pathogenesis and suggest novel therapeutic approaches targeting both immune cells and plasma metabolites.

## Methodologies and materials

### Study design

This study employed a bidirectional MR analysis to investigate the CR between immune cells and CP and to explore the potential mediating role of plasma metabolites through mediation analysis. In this framework, CP was defined as the primary outcome, with immune cells considered as potential exposure factors, allowing for a comprehensive examination of the CR between immune cells and CP. Additionally, we assessed plasma metabolites as possible mediators in the immune cell-CP relationship, examining the extent to which these metabolites contribute to this relationship.

### Source of chronic pancreatitis GWAS data

The GWAS data for CP were obtained from a large-scale meta-analysis conducted by Sakaue et al. [18]. This analysis included samples from 1,424 CP patients and 476,104 healthy individuals of European descent and utilized a total of 24,195,431 SNP loci for the association analysis. The data are publicly accessible at the website (10.1038/s41588-021-00931-x).

### Source of immune cell GWAS data

Genetic association data for immune cells were sourced from a cohort study involving 3,757 individuals of European descent [19]. These datasets, accessible via the IEU Open GWAS platform (https://gwas.mrcieu.ac.uk), encompass identifiers from ebi-a-GCST0001391 to ebi-a-GCST0002121. The study includes 192 measures of relative cell counts, 32 parameters of cell morphology, 118 measures of absolute cell counts and 389 median fluorescence intensity readings reflecting surface antigen levels.

### Source of plasma metabolite GWAS data

The GWAS dataset for plasma metabolites was obtained from a Canadian longitudinal aging study [20], involving 8,299 participants, which provided genetic information on 1,400 metabolites. The summary data from this study are accessible through the GWAS database (https://www.ebi.ac.uk/gwas), with identifiers ranging from GCST90199621 to GCST90201020.

### Instrumental variable (IV) screening

Unified inclusion and exclusion criteria were applied to the genetic variation of 731 immune cells and 1,400 plasma metabolites. Prior research indicates that using a genome-wide significance threshold of *P* < 5 × 10⁻⁸ yields very few SNPs closely linked to immune cells and plasma metabolites, which is insufficient for robust analysis. Therefore, this study adopted a threshold of *P* < 1 × 10⁻⁵, a commonly used criterion in MR analyses [21].

After identifying significant SNPs for each metabolite, linkage disequilibrium analysis was conducted. SNPs were retained as independent genetic variations if they met the following three conditions: (1) located on the same chromosome, (2) within 10,000 kb, and (3) with a linkage disequilibrium parameter (r² > 0.001). To ensure a robust association between the IVs and exposure factors, we used the F-statistic to assess the strength of the IVs; an F-value below 10 indicates weak IVs, which could introduce bias in causal inference.

To further mitigate the impact of confounding factors on the IVs, we used the PhenoScanner database to exclude SNPs associated with factors strongly linked to CP, such as alcohol consumption, smoking, BMI, and diabetes [22].

### Mediation analysis

The mediating analysis involves a two-step methodology. Firstly, we calculate the causative influence of immune cells on plasma metabolites (β1). Then, we assess the causative influence of plasma metabolites on CP (β2). The total causative influence of immune cells on CP is represented as β, while the direct effect is denoted as β0. The fraction of the mediating effect is indicated as R and is calculated as R = $\:\frac{{\beta}1\text{*}{\beta}2}{{\beta}}$ The direct effect of immune cells on CP is given by β0 = β – (β1*β2).

### Statistical analysis

The statistical examination was executed via R software (version 4.3.1) with the TwoSampleMR (version 0.6.4) and MRPRESSO (version 1.0) packages. This study used five MR analysis methods - inverse variance weighted (IVW), weighted median, simple mode, weighted mode, and MR Egger - to evaluate the CR between exposure and outcomes. Due to the robustness of IVW in handling multiple instrumental variables and its ability to provide accurate estimates when assuming non pleiotropy and sufficient instrument strength, it served as the primary reference method.It is essential to verify that the IVs do not affect the result via elements unconnected to the exposure variable to uphold the integrity of the independence and exclusivity assumptions. The MR-Egger intercept assessment was employed to evaluate horizontal pleiotropy, ensuring the reliability of the study results. A *P*-value exceeding 0.05 suggested the lack of horizontal pleiotropy [23]. Additionally, we used Cochran’s Q statistic to quantitatively evaluate the heterogeneity among the chosen IVs, where a *P*-value exceeding 0.05 suggests a lack of heterogeneity [24]. Additionally, a leave-one-out sensitivity analysis was executed to evaluate the impact of each SNP on the MR analysis results [25]. Finally, the results were visually represented using scatter plots, forest plots, leave-one-out plots, and funnel plots.

## Results

### Investigating the overall causal impact of immune cells on CP

Using the three GWAS summary datasets, we identified and incorporated IVs based on predetermined significance thresholds (see Supplementary Tables 1–3). We then used 731 immune cell types as exposures and CP as the outcome in a two-sample MR analysis, applying five different methods to explore CRs between immune cells and CP. We examined the consistency of directional effects, assessed horizontal pleiotropy, and evaluated heterogeneity across these methods, ultimately identifying seven immune cells causally related to CP.

According to the IVW method, the ORs for CD25 on IgD^−^ CD38^−^ (OR = 1.105, 95% CI = 1.035–1.180, *P* = 0.003), CD19 on IgD^+^ CD24^+^ (OR = 1.076, 95% CI = 1.019–1.136, *P* = 0.008), CD4^+^ % T cell (OR = 1.156, 95% CI = 1.039–1.287, *P* = 0.008), and CD24 on transitional B cells (OR = 1.177, 95% CI = 1.043–1.328, *P* = 0.008) were all greater than 1, indicating that these are risk factors for CP.

**Fig. 1:**
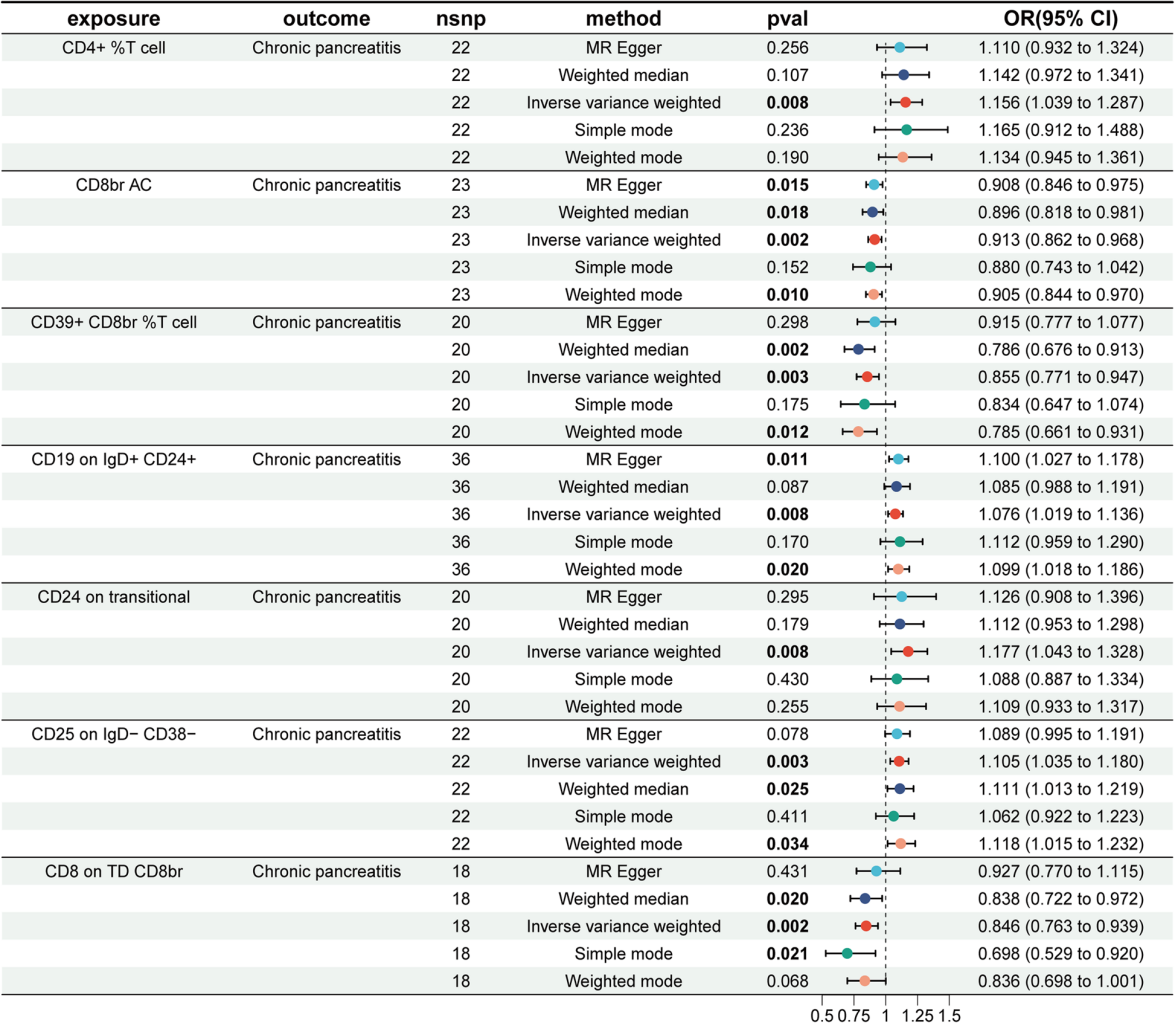
Forest plot showing the causal effect of seven immune cells on CP

Conversely, the ORs for CD8 on TD CD8br (OR = 0.846, 95% CI = 0.763–0.939, *P* = 0.002), CD39^+^ CD8br %T cell (OR = 0.855, 95% CI = 0.771–0.947, *P* = 0.003), and CD8br AC (OR = 0.913, 95% CI = 0.862–0.968, *P* = 0.002) were all less than 1, indicating these cells act as protective factors against CP. The forest plot in Fig. 1 presents the outcomes of the five MR analysis methods, with the seven immune cells as exposures and CP as the outcome. Detailed MR analysis findings are shown in Supplementary Table 4.

The MR-Egger and MR-PRESSO tests for horizontal pleiotropy indicated no evidence of pleiotropy, as *P*-values for all seven immune cells exceeded 0.05 (Supplementary Table 5), supporting the reliability of these results. Additionally, heterogeneity analysis using MR-Egger and IVW methods showed no heterogeneity for the seven immune cells, with all *P*-values above 0.05 (Supplementary Table 6).

For further insights, Supplementary Figs. 1–4 provide scatter plots, funnel plots, leave-one-out plots, and individual forest plots, respectively. These findings collectively suggest that immune cells contribute to the development of CP.

### Exploring the causal impact of metabolite levels on CP

**Fig. 2:**
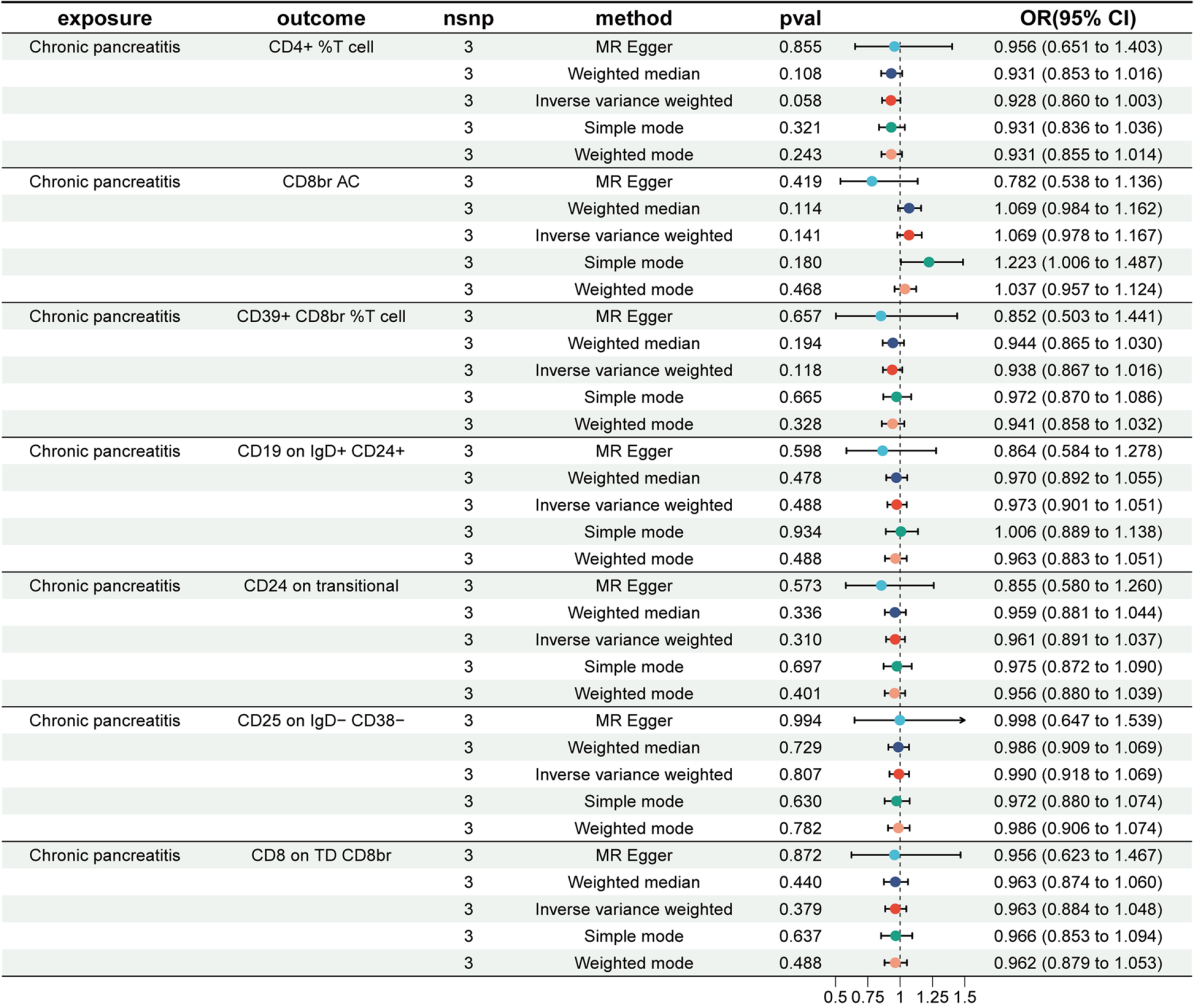
Forest plot showing the causal effect of CP on seven immune cells

To meet the criteria for mediation MR analysis, we conducted a reverse MR analysis by treating CP as the exposure variable and the seven immune cells as outcome variables, using five different MR methods. The MR results indicated no reverse CRs between CP and the seven immune cells, as all *P*-values exceeded 0.05. The forest plot in Fig. 2 illustrates the results of the MR analysis, with CP as the exposure and the seven immune cells as outcomes.

### Exploring the causal impact of plasma metabolite levels on CP

In a subsequent analysis, a two-sample MR approach was used to examine 1,400 plasma metabolites as exposures and CP as the outcome. This analysis identified CRs between 12 metabolites and CP. According to the IVW method, the following metabolites were found to be risk factors for CP, with ORs greater than 1: 1-palmitoyl-2-dihomo-linolenoyl-GPC (16:0/20:3n3 or 6) (OR = 1.243, 95% CI = 1.090–1.417, *P* = 0.001), docosatrienoate (22:3n3) (OR = 1.256, 95% CI = 1.082–1.458, *P* = 0.003), 1-(1-enyl-palmitoyl)−2-linoleoyl-GPE (p-16:0/18:2) (OR = 1.310, 95% CI = 1.100–1.561, *P* = 0.003), and cholesterol (OR = 1.334, 95% CI = 1.123–1.584, *P* = 0.001).

Conversely, the following metabolites had ORs less than 1, indicating they serve as protective factors against CP: Trigonelline (OR = 0.580, 95% CI = 0.444–0.758, *P* = 6.57E-05), myo-inositol (OR = 0.744, 95% CI = 0.629–0.882, *P* = 0.0006), 5alpha-androstan-3alpha,17beta-diol monosulfate (2) (OR = 0.780, 95% CI = 0.651–0.934, *P* = 0.007), adenosine 5’-monophosphate (AMP) to cysteine ratio (OR = 0.810, 95% CI = 0.711–0.923, *P* = 0.001), glycerol to carnitine ratio (OR = 0.831, 95% CI = 0.728–0.947, *P* = 0.006), 1-arachidonoyl-GPC (20:4n6) (OR = 0.849, 95% CI = 0.772–0.933, *P* = 0.0007), flavin adenine dinucleotide (FAD) (OR = 0.859, 95% CI = 0.769–0.959, *P* = 0.007), and 1-palmitoyl-2-arachidonoyl-GPC (16:0/20:4n6) (OR = 0.871, 95% CI = 0.797–0.951, *P* = 0.002).

**Fig. 3:**
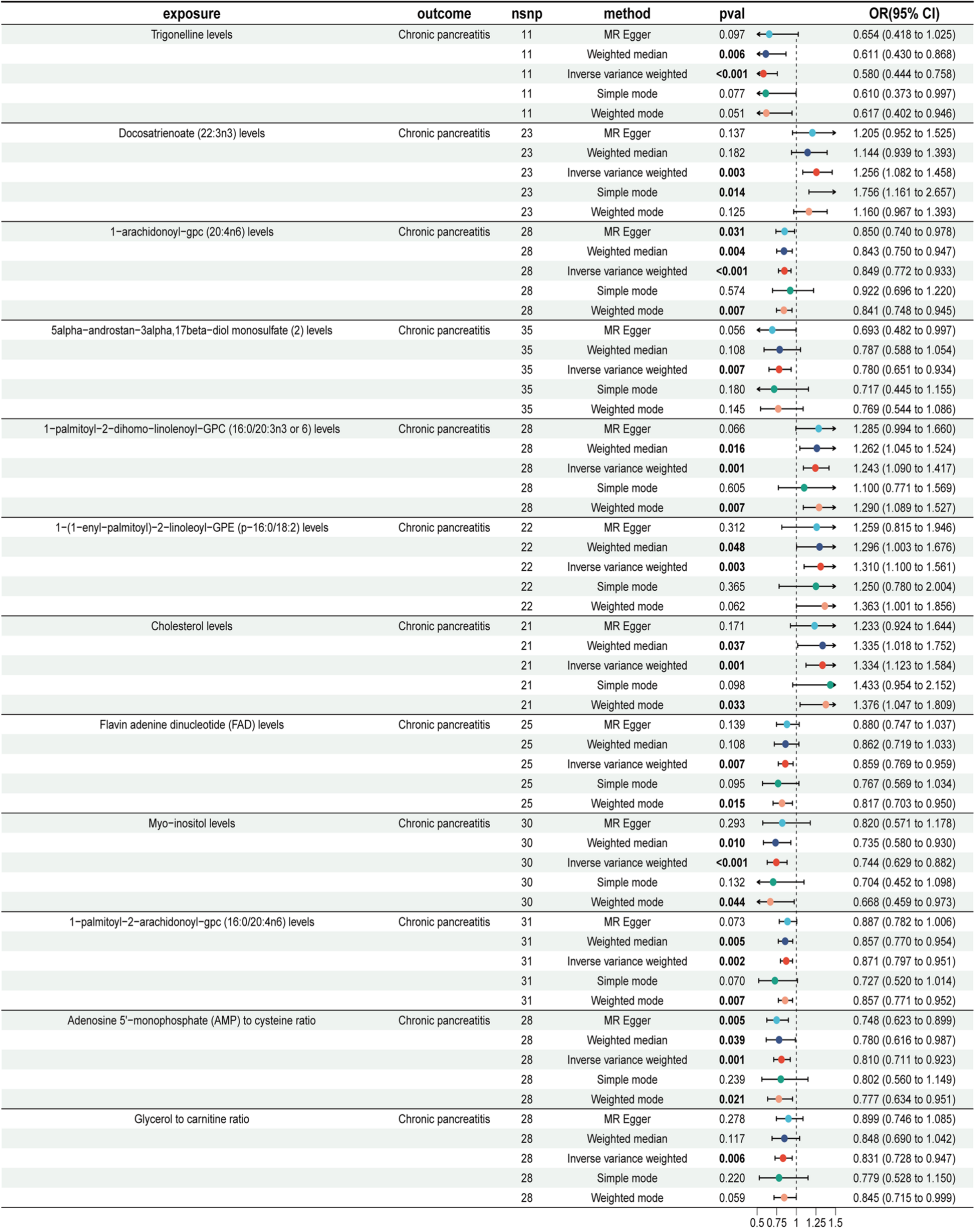
Forest plot showing the causal effect of twelve metabolite levels on CP

The forest plot in Fig. 3 presents the MR analysis results with these 12 metabolites as exposures and CP as the outcome. Supplementary Table 7 provides detailed MR findings. The MR-Egger and MR-PRESSO tests for pleiotropy showed *P*-values exceeding 0.05 for all 12 metabolites, suggesting no pleiotropy (Supplementary Table 8). Heterogeneity analyses using MR-Egger and IVW methods found no evidence of heterogeneity in any of the 12 metabolites (Supplementary Table 9). For additional details, Supplementary Figs. 5–8 offer scatter plots, funnel plots, leave-one-out plots, and individual forest plots, respectively. These findings indicate that plasma metabolites influence CP development.

### Exploring the causal impact of immune cells on metabolite levels

After identifying immune cells and metabolite levels that exhibited causal relationships with chronic pancreatitis, a two-sample MR analysis was performed. Since no reverse causal relationships were detected in the second MR step, seven immune cell types from the initial step were selected for mediation MR analysis. The findings revealed causal links between CD25 on IgD^−^ CD38^−^ cells and cholesterol levels, CD8br AC cells and the glycerol-to-triglyceride ratio, and CD8 on TD CD8br cells and FAD levels.

According to the IVW method, the OR for the relationship between CD25 on IgD − CD38 − and cholesterol levels was 1.032 (95% CI = 1.002–1.064, *P* = 0.036). The OR for CD8 on TD CD8br and FAD levels was 0.942 (95% CI = 0.895–0.993, *P* = 0.025). The OR for CD8br AC and the glycerol-to-triglyceride ratio was 0.973 (95% CI = 0.945–0.999, *P* = 0.040). Notably, the direction of these effects was consistent across the five MR analysis methods, with ORs either consistently below or above 1 for all three CRs.

**Fig. 4:**
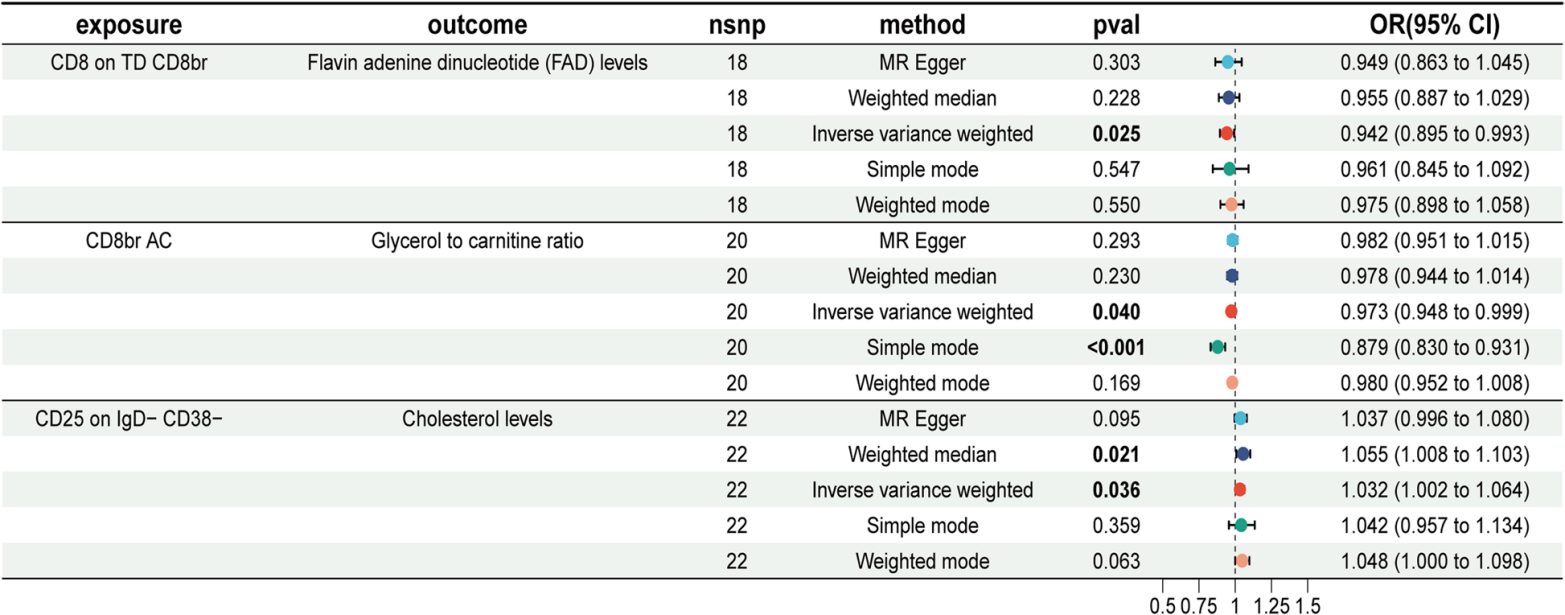
Forest plot showing the causal effects of the three immune cell types on the three metabolite levels

Figure 4 presents a forest plot illustrating the results of these MR analyses, with the three immune cells as exposures and the three metabolite levels as outcomes. Detailed MR analysis results are available in Supplementary Table 10. The MR-Egger and MR-PRESSO tests for pleiotropy showed *P*-values above 0.05 for all three immune cells, indicating no pleiotropy (Supplementary Table 11). Heterogeneity analyses using MR-Egger and IVW approaches also revealed no evidence of heterogeneity, with all *P*-values exceeding 0.05 (Supplementary Table 12). Supplementary Figs. 9–12 provide additional insights, including scatter plots, funnel plots, leave-one-out plots, and individual forest plots. These findings suggest that immune cells influence plasma metabolite levels.

### Genetically predicted cholesterol levels mediate the link between CD25 on IgD−CD38− and CP

**Fig. 5:**
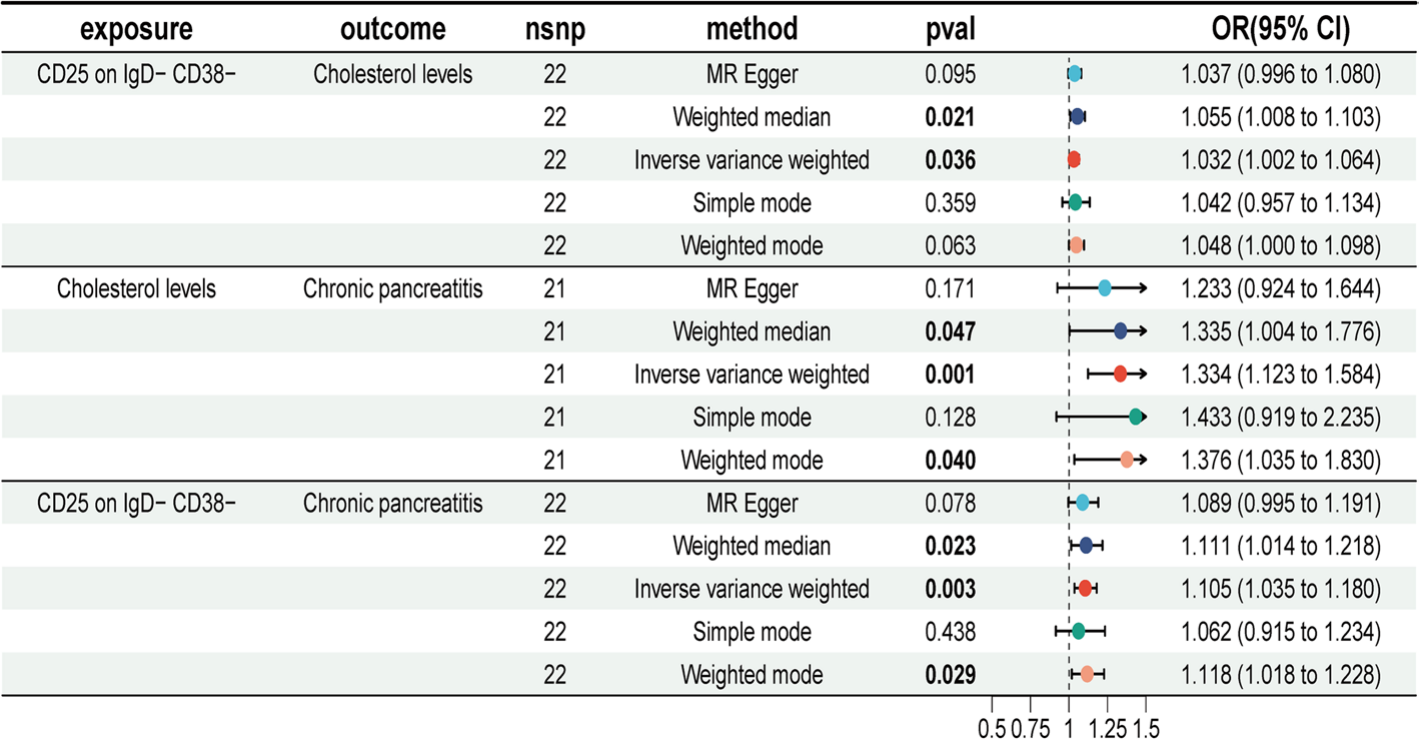
Forest plot of the mediation MR analysis

**Fig. 6:**
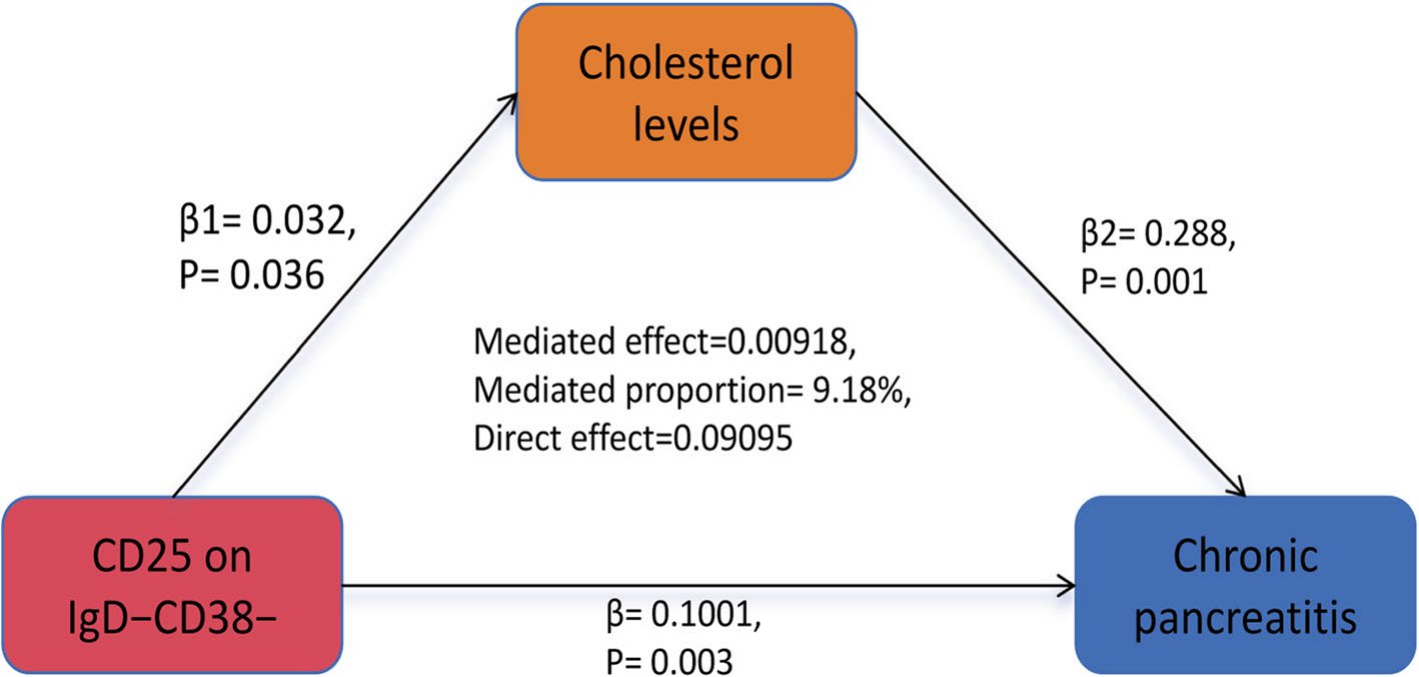
Schematic representation of the results from the mediation MR analysis

In the final analysis, we summarized the results of the mediation MR analysis to identify the mediator. Figure 5 displays the aggregated forest plot, and Fig. 6 illustrates the schematic of the mediation MR analysis. CD25 on IgD^−^CD38^−^ was identified as a risk factor for elevated cholesterol levels (b = 0.032, *P* = 0.036), and cholesterol levels were subsequently found to be a risk factor for CP (b = 0.288, *P* = 0.001). Additionally, CD25 on IgD^−^CD38^−^ was directly associated with CP risk (b = 0.1001, *P* = 0.003). The mediation effect was calculated as 0.00918, accounting for 9.18% of the total effect, while the direct effect was 0.09095, representing 90.82% of the total effect. These findings indicate that CD25 on IgD^−^CD38^−^ cells contribute to the development of CP by elevating cholesterol levels.

## Discussion

Immune cells are key contributors to both the onset and progression of CP [8]; however, the intricate interactions between immune cells and chronic pancreatitis are not yet fully understood. Metabolic disturbances are a hallmark of CP [26], and recent evidence indicates a possible interplay between immune cells and plasma metabolites [27, 28]. The difficulty in obtaining tissue samples from CP patients has hindered the discovery and validation of immune cell functions. In this study, we used a two-step MR approach to investigate whether plasma metabolites mediate the CR between immune cells and CP. Our findings indicate that four immune cells and four metabolites serve as risk factors for CP, while three immune cells and eight metabolites act as protective factors. Notably, our study identified CD25 on IgD^−^ CD38^−^ B cells as promoting CP development by increasing cholesterol levels. This discovery has the potential to enhance our understanding of CP pathogenesis and may provide valuable insights for developing future therapies targeting immune cells and plasma metabolites.

Our investigation indicated that elevated levels of CD25 on IgD^−^ CD38^−^ cells, CD24 on transitional B cells, CD19 on IgD^+^ CD24 + B cells, and CD4^+^ % T cells may be associated with the development of CP. CD25 on IgD^−^ CD38^−^ is part of the B cell panel, and there are currently no published studies on this specific cell subtype. Since this B cell lacks both IgD and CD38, it is thought to represent either immature or transitional B cells. Both CD24 on transitional B cells and CD19 on IgD^+^ CD24^+^ B cells fall within the transitional B cell subset. Previous studies have shown that transitional and immature B cells can produce IL-6, which regulates the proliferation of CD4^+^ T cells and their differentiation into T helper (Th) effector cells [29]. CD4^+^ % T cells, also known as CD4^+^ T cells, play a critical role in regulating immune responses. During the acute inflammatory phase of pancreatitis, activated Th1 cells stimulate other effector immune cells, such as B cells and CD8^+^ cytotoxic T lymphocytes, leading to pancreatic damage, which can be mitigated by depleting CD4^+^ T cells [30, 31]. Conversely, in the chronic inflammatory phase, Th2 cell activation becomes the predominant pathway for CD4^+^ T cell differentiation, which activates macrophages and pancreatic stellate cells, aiding in pancreatic repair and contributing to pathological fibrosis [8]. This suggests that immature B cells, transitional B cells, and CD4^+^ T cells may have critical regulatory functions in the immune response of CP patients. However, further studies are needed to confirm the roles of these cells in CP.

In contrast, our findings suggest that a higher proportion of CD39^+^ CD8br % T cells, CD8 on TD CD8br cells, and CD8br AC cells may reduce the risk of CP. CD39^+^ CD8br % T cells belong to the Treg cell panel, while CD8 on TD CD8br and CD8br AC cells are within the CD8^+^ T cell panel. CP is characterized by a type 2 immune response involving Th2 cells and macrophages [32, 33]. In CP, Treg cells limit conventional T cell proliferation by secreting IL-10, thereby suppressing the type 2 immune response [34]. Additionally, studies have shown that specifically knocking out Treg cells in CP mouse models significantly worsens fibrosis and exocrine dysfunction in experimentally induced CP [35]. These findings suggest that CD39^+^ CD8br % T cells may help mitigate inflammatory responses, potentially playing a protective role in CP. In CP tissues, alongside Treg cells, a substantial population of CD8^+^ T cells is present, although their exact functions remain largely unknown [10]. Interestingly, experimental studies on fibrosis in nonalcoholic steatohepatitis have demonstrated that adoptively transferred CD8^+^ T cells can induce apoptosis in hepatic stellate cells, promoting the resolution of liver fibrosis [36]. Thus, we hypothesize that CD8^+^ T cells may protect against CP by inducing apoptosis in pancreatic stellate cells, thereby reducing pancreatic fibrosis. However, the precise functions and mechanisms of CD8^+^ T cells in chronic pancreatitis require further investigation.

Our study suggests that high cholesterol levels contribute to CP risk. A retrospective study identified hypertriglyceridemia as the most common cause of recurrent acute pancreatitis, and elevated low-density lipoprotein (LDL) cholesterol as an independent risk factor for this condition [37]. Another retrospective case-control study found a positive association between serum amylase and LDL cholesterol levels in individuals with CP. Additionally, it revealed that urinary amylase levels were positively linked to both total cholesterol and LDL cholesterol, indicating that total cholesterol also contributes to CP risk [38]. These findings align with our study, suggesting that increased cholesterol levels are a risk factor for CP. Notably, the mediation MR analysis revealed a CR between CD25 on IgD^−^CD38^−^ cells and cholesterol levels, suggesting that CD25 on IgD^−^CD38^−^ cells may contribute to CP development by increasing cholesterol levels. This finding underscores the interplay between immune cells and metabolites in CP pathogenesis, offering valuable insights for future research.

Conversely, our study indicates that certain metabolites and metabolic ratios may protect against CP. These include trigonelline, myo-inositol, 5alpha-androstan-3alpha,17beta-diol monosulfate, the AMP-to-cysteine ratio, FAD, 1-arachidonoyl-GPC, 1-palmitoyl-2-arachidonoyl-GPC, and the glycerol-to-carnitine ratio. Pancreatitis is often characterized by lipid metabolism dysregulation, with high triglyceride levels playing a significant role [38]. Trigonelline, a naturally occurring alkaloid, stimulates the p38 MAPK/ATF-2 signaling pathway by activating β3-adrenergic receptors and inhibiting phosphodiesterase 4, reducing lipogenesis while promoting lipolysis and fatty acid oxidation [39]. Myo-inositol, a cyclic polyol, is known to promote the secretion of thyroid hormones and insulin. It has been shown to reduce fatty acid synthesis by regulating lipid synthesis transcription factors, thereby lowering plasma triglycerides [40]. 5alpha-androstan-3alpha,17beta-diol monosulfate, a metabolite derived from the reduction of dihydrotestosterone, acts as an agonist of the SHBG (sex hormone-binding globulin) receptor, increasing intracellular cAMP levels and promoting long-chain fatty acid oxidation [41, 42]. AMP, or adenosine monophosphate, activates AMP-activated protein kinase (AMPK), which stimulates fatty acid oxidation and ATP synthesis [43]. Cysteine dioxygenase 1 (Cdo1), essential for taurine synthesis from cysteine, has been linked to lipolytic capacity in adipose tissue, with *Cdo1* knockout mice displaying reduced free fatty acids and increased susceptibility to obesity [44]. FAD is a coenzyme involved in fatty acid β-oxidation, facilitating electron transfer and lowering blood lipids [45]. Integrating these insights with our findings, we propose that trigonelline, AMP, FAD, and myo-inositol could serve as potential targets for future CP therapies.

Our study also revealed that certain metabolites, such as 1-palmitoyl-2-dihomo-linolenoyl-GPC, 1-(1-enyl-palmitoyl)−2-linoleoyl-GPE, 1-arachidonoyl-GPC, and docosatrienoate, are associated with increased CP risk, while 1-palmitoyl-2-arachidonoyl-GPC appears protective. These metabolites are derived from glycerophospholipids and contain various polyunsaturated fatty acids (PUFAs) like arachidonic, palmitic, and linoleic acids. Although dietary supplementation with PUFAs is known to lower plasma triglycerides [46, 47] our findings differ from previous PUFA studies, possibly due to structural variations among glycerophospholipid subclasses that may lead to distinct biological effects [48].

Additionally, our findings suggest that the glycerol-to-carnitine ratio may be protective against CP. Glycerol, primarily produced in the small intestine and liver, is converted into triglycerides and stored for energy release when needed [49]. Elevated glycerol promotes triglyceride synthesis, raising blood lipid levels. Carnitine, an essential coenzyme for acyltransferases, facilitates fatty acid β-oxidation and lowers blood lipids [50]. While our findings differ from previous studies on these metabolites, they highlight the need for further research to better understand these relationships in CP.

This study has several strengths and limitations. First, a major strength is the use of large-scale GWAS data and multiple MR analysis methods. To our knowledge, this is the first comprehensive investigation of the role of adaptive immune cells in CP progression and the potential mediating effects of plasma metabolites. Additionally, this study improves our understanding of CP pathogenesis and offers new insights for future therapies targeting immune cells and metabolites. However, there are limitations to consider. Our dataset primarily includes European populations and lacks representation from other demographic groups, highlighting the need for future research to incorporate diverse populations. Moreover, we applied a relatively lenient threshold (*P* < 1 × 10⁻⁵) for IV selection, which may have increased the heterogeneity of these variables. Finally, as this study is limited to genetic association analyses, further foundational experiments and clinical studies are necessary to validate these findings.

In conclusion, this study has uncovered causal connections among immune cells, plasma metabolites, and CP. The findings suggest that certain immune cell markers, including CD25 on IgD^−^ CD38^−^ B cells, CD19 on IgD^+^ CD24^+^ B cells, CD4^+^ % T cells, and CD24 on transitional B cells, are associated with an elevated risk of CP, while markers such as CD8 on TD CD8br, CD39^+^ CD8br % T cells, and CD8br AC cells appear to offer protection against CP. Additionally, specific plasma metabolites—such as 1-palmitoyl-2-dihomo-linolenoyl-GPC, docosatrienoate, and 1-(1-enyl-palmitoyl)−2-linoleoyl-GPE—were identified as risk factors for CP. Conversely, trigonelline, myo-inositol, 5alpha-androstan-3alpha,17beta-diol monosulfate, the AMP-to-cysteine ratio, and FAD exhibited protective effects. Importantly, mediation MR analysis revealed that CD25 on IgD^−^ CD38^−^ B cells may promote CP development by increasing cholesterol levels. This study sheds light on the complex interactions between immune cells and metabolites in CP pathogenesis, offering new insights and potential targets for future clinical research.

## Supplementary Information

Supplementary Material 1.

Supplementary Material 2.

## Data Availability

Data is provided within the manuscript or supplementary information files.

## Abbreviations

CP: Chronic Pancreatitis
MR: Mendelian Randomization
OR: Odds Ratio
IVs: Instrumental Variables
IVW: Inverse Variance Weighted
AMP: Adenosine 5’-Monophosphate
FAD: Flavin Adenine Dinucleotide
Th: T helper
Tregs: regulatory T cells
AMPK: AMP-Activated Protein Kinase
Cdo1: Cysteine dioxygenase 1
LDL: Low-Density Lipoprotein
PUFAs: Polyunsaturated Fatty Acids
