# Supplementary figures and images for: Causal roles of immune cells and metabolites in chronic pancreatitis: a mendelian randomization study
Source: Hereditas. 2025 Feb 12;162:20. doi: 10.1186/s41065-025-00378-8 (PMC11816568; doi:10.1186/s41065-025-00378-8)

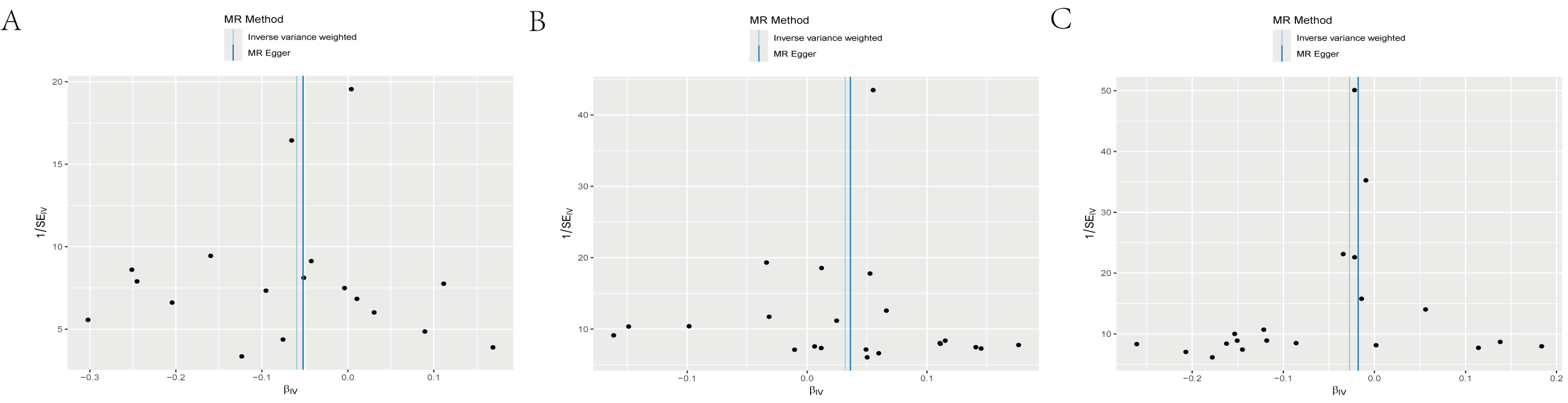

Supplement: Supplementary file 2 — Supplementary Material 2. [file 41065_2025_378_MOESM2_ESM.zip › Supplementary Figure 10.tif]

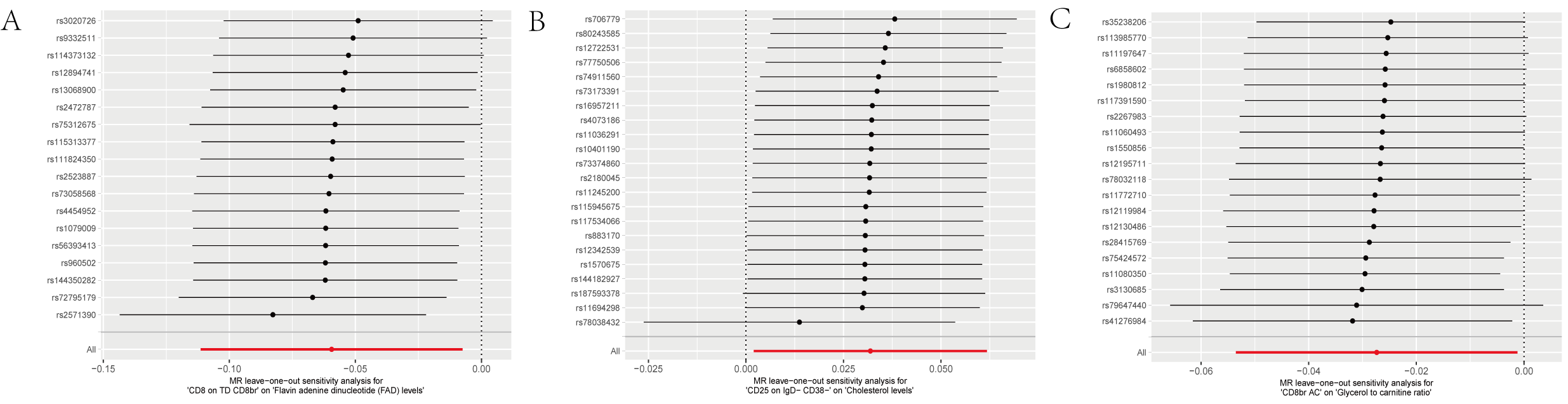

Supplement: Supplementary file 2 — Supplementary Material 2. [file 41065_2025_378_MOESM2_ESM.zip › Supplementary Figure 11.tif]

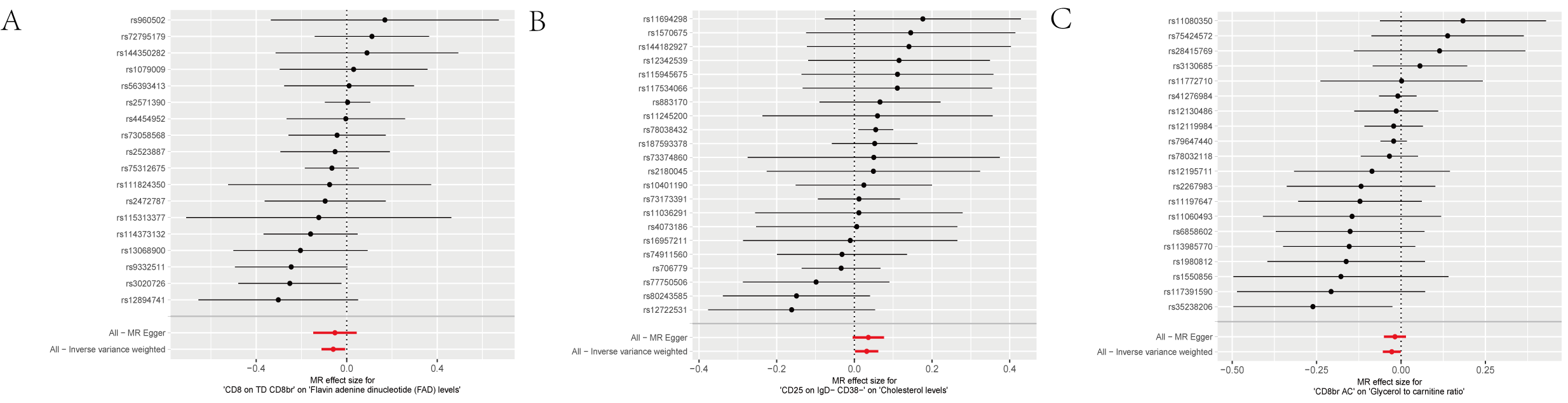

Supplement: Supplementary file 2 — Supplementary Material 2. [file 41065_2025_378_MOESM2_ESM.zip › Supplementary Figure 12.tif]

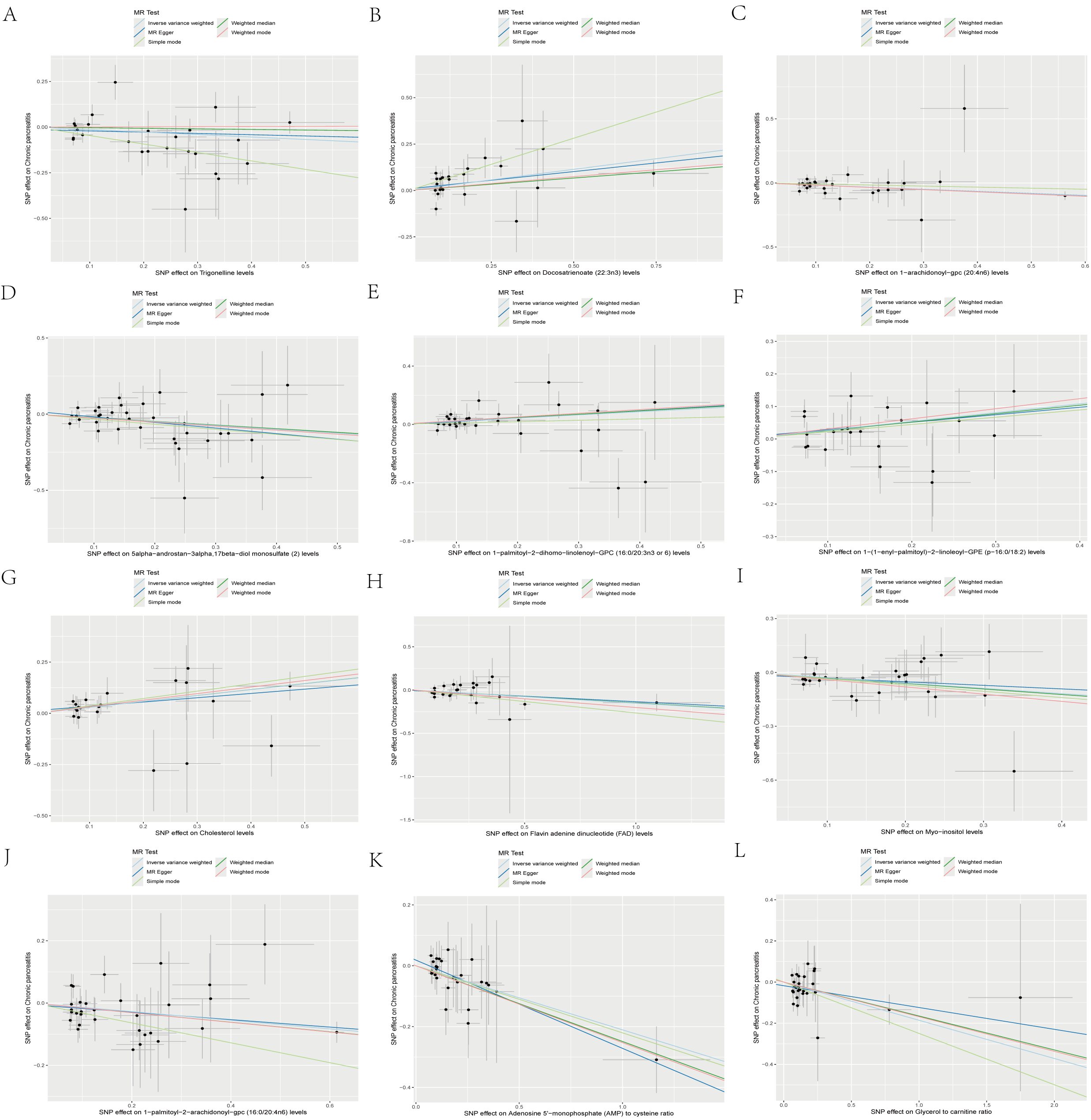

Supplement: Supplementary file 2 — Supplementary Material 2. [file 41065_2025_378_MOESM2_ESM.zip › Supplementary Figure 5.tif]

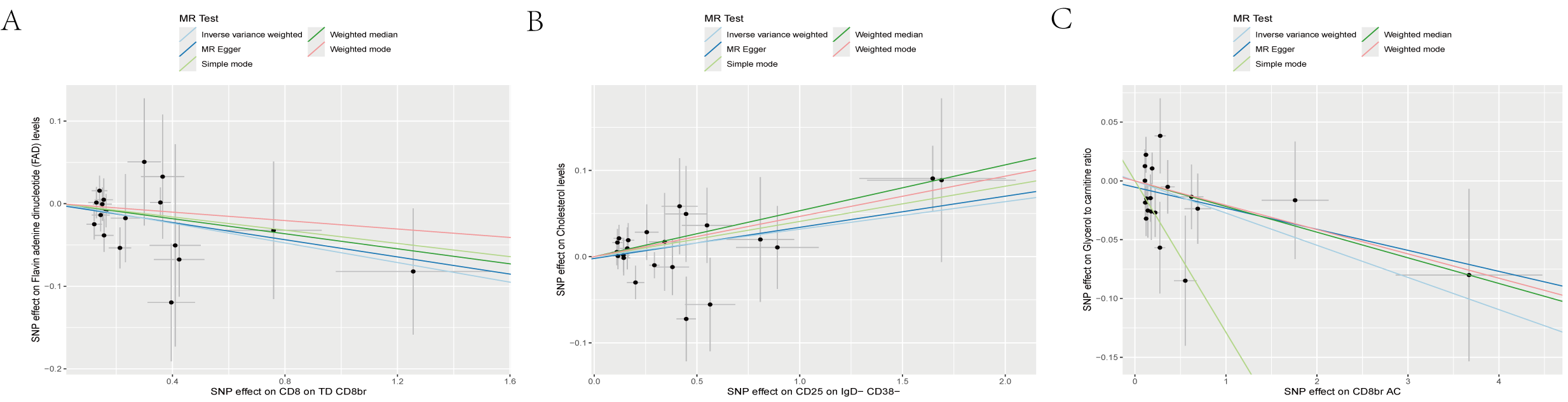

Supplement: Supplementary file 2 — Supplementary Material 2. [file 41065_2025_378_MOESM2_ESM.zip › Supplementary Figure 9.tif]

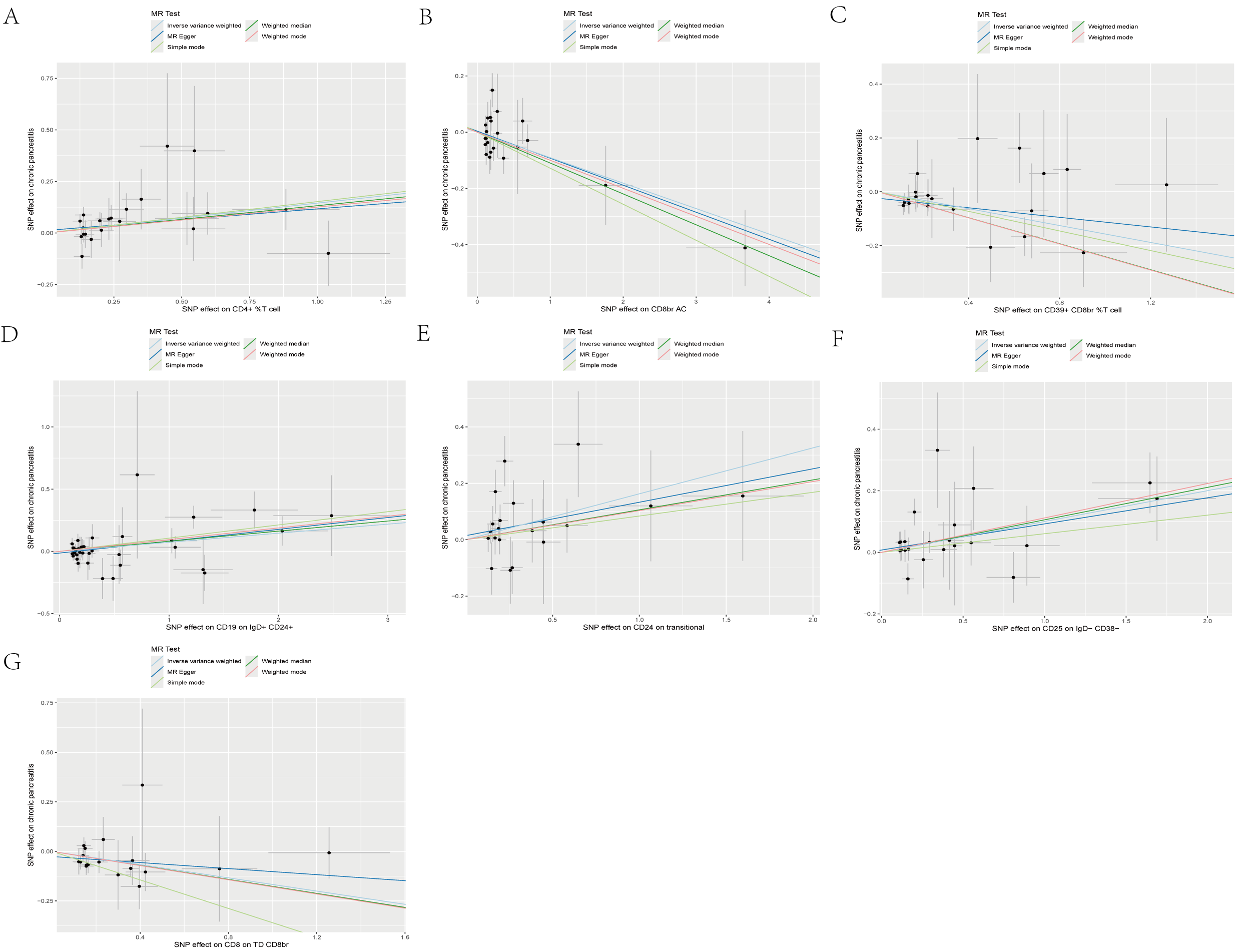

Supplement: Supplementary file 2 — Supplementary Material 2. [file 41065_2025_378_MOESM2_ESM.zip › Supplementary Figure 1.tif]

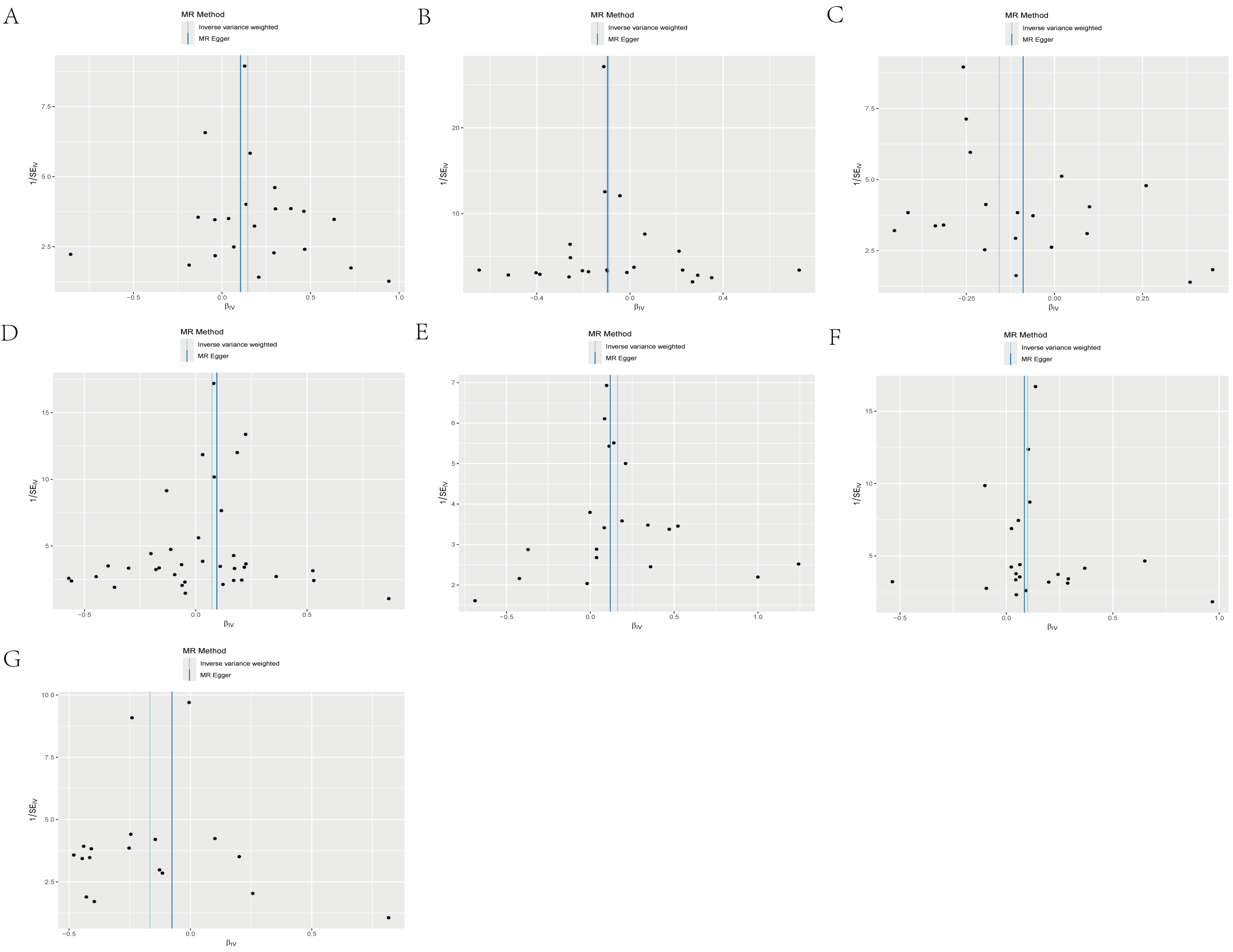

Supplement: Supplementary file 2 — Supplementary Material 2. [file 41065_2025_378_MOESM2_ESM.zip › Supplementary Figure 2.tif]

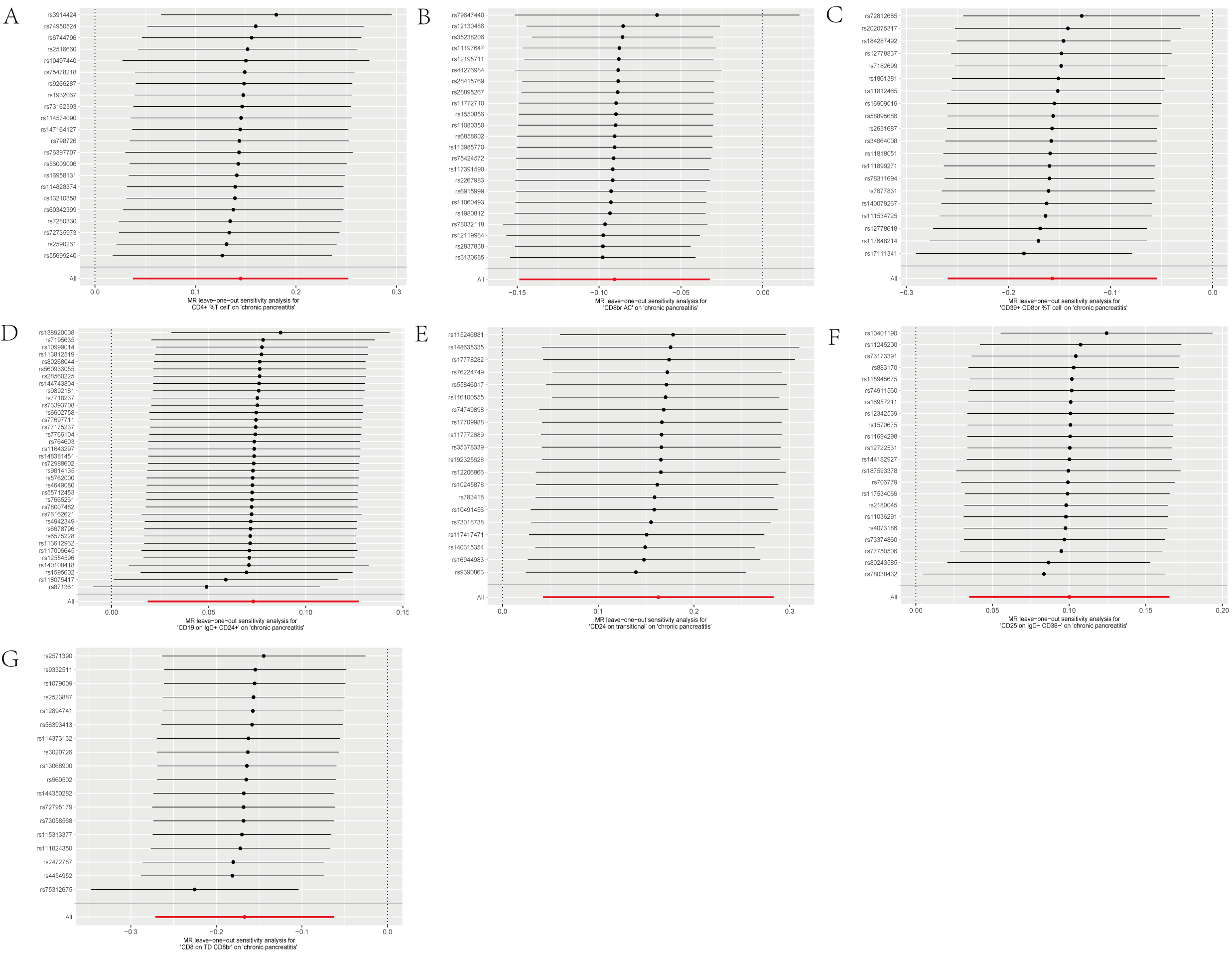

Supplement: Supplementary file 2 — Supplementary Material 2. [file 41065_2025_378_MOESM2_ESM.zip › Supplementary Figure 3.tif]

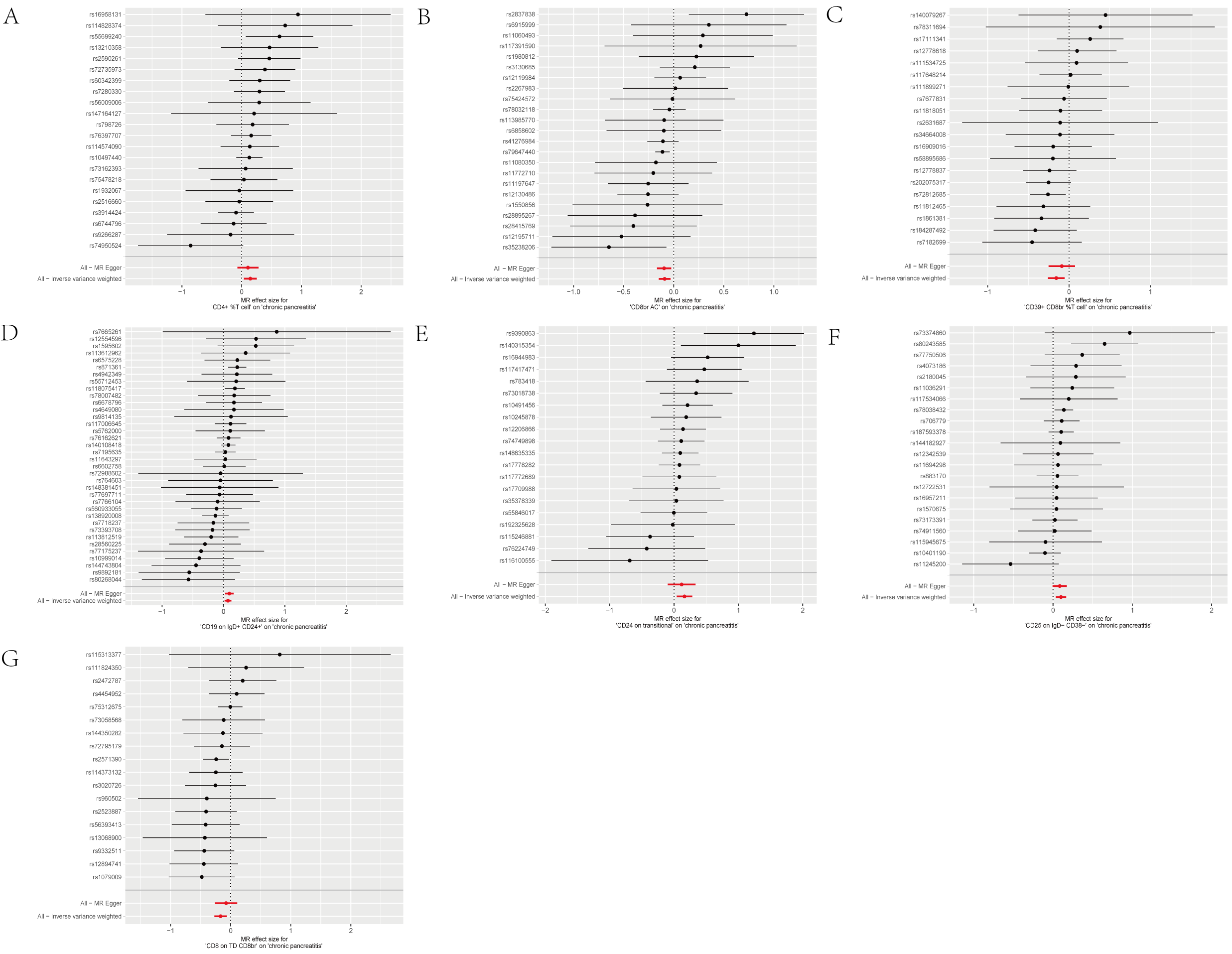

Supplement: Supplementary file 2 — Supplementary Material 2. [file 41065_2025_378_MOESM2_ESM.zip › Supplementary Figure 4.tif]

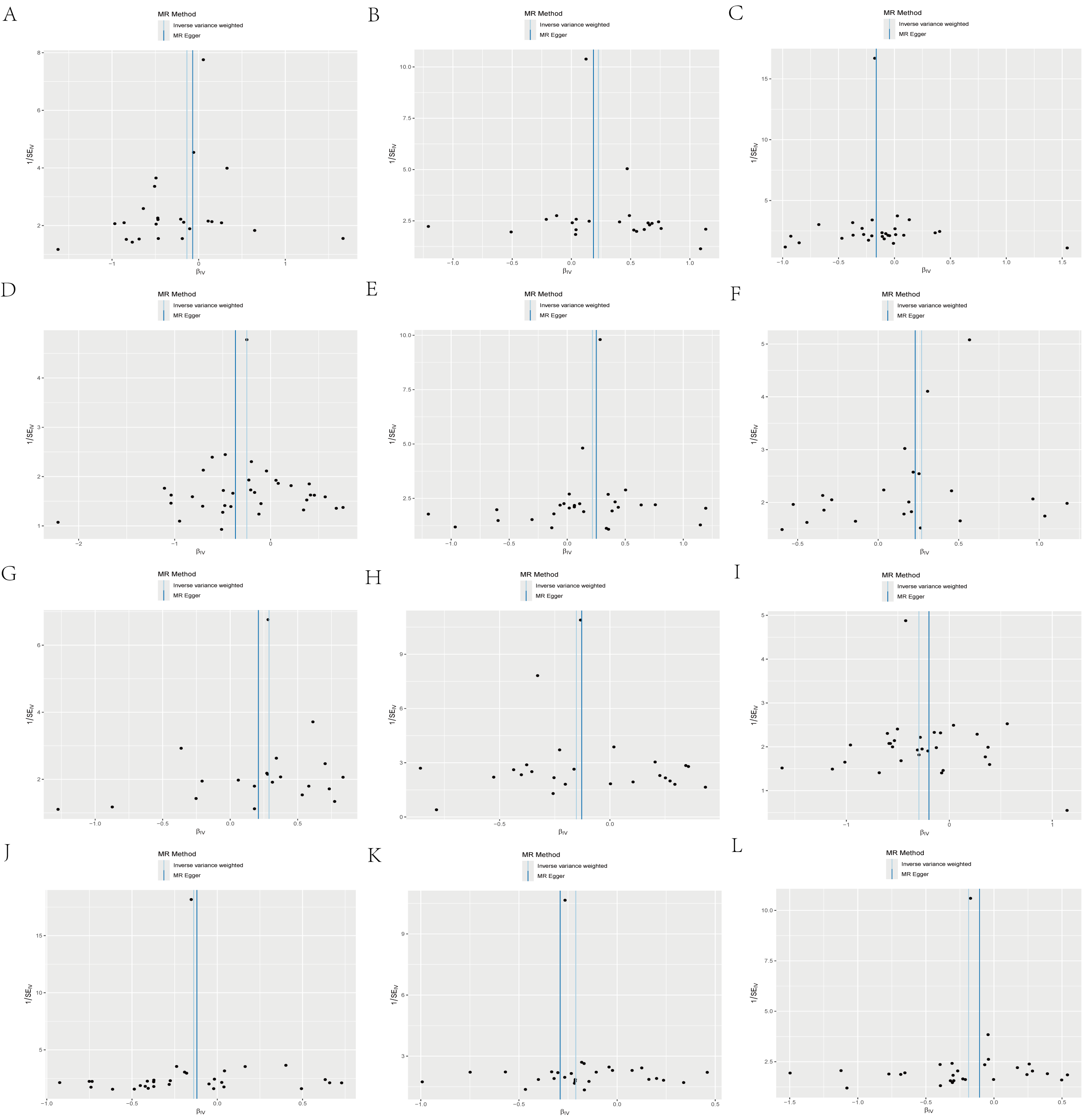

Supplement: Supplementary file 2 — Supplementary Material 2. [file 41065_2025_378_MOESM2_ESM.zip › Supplementary Figure 6.tif]

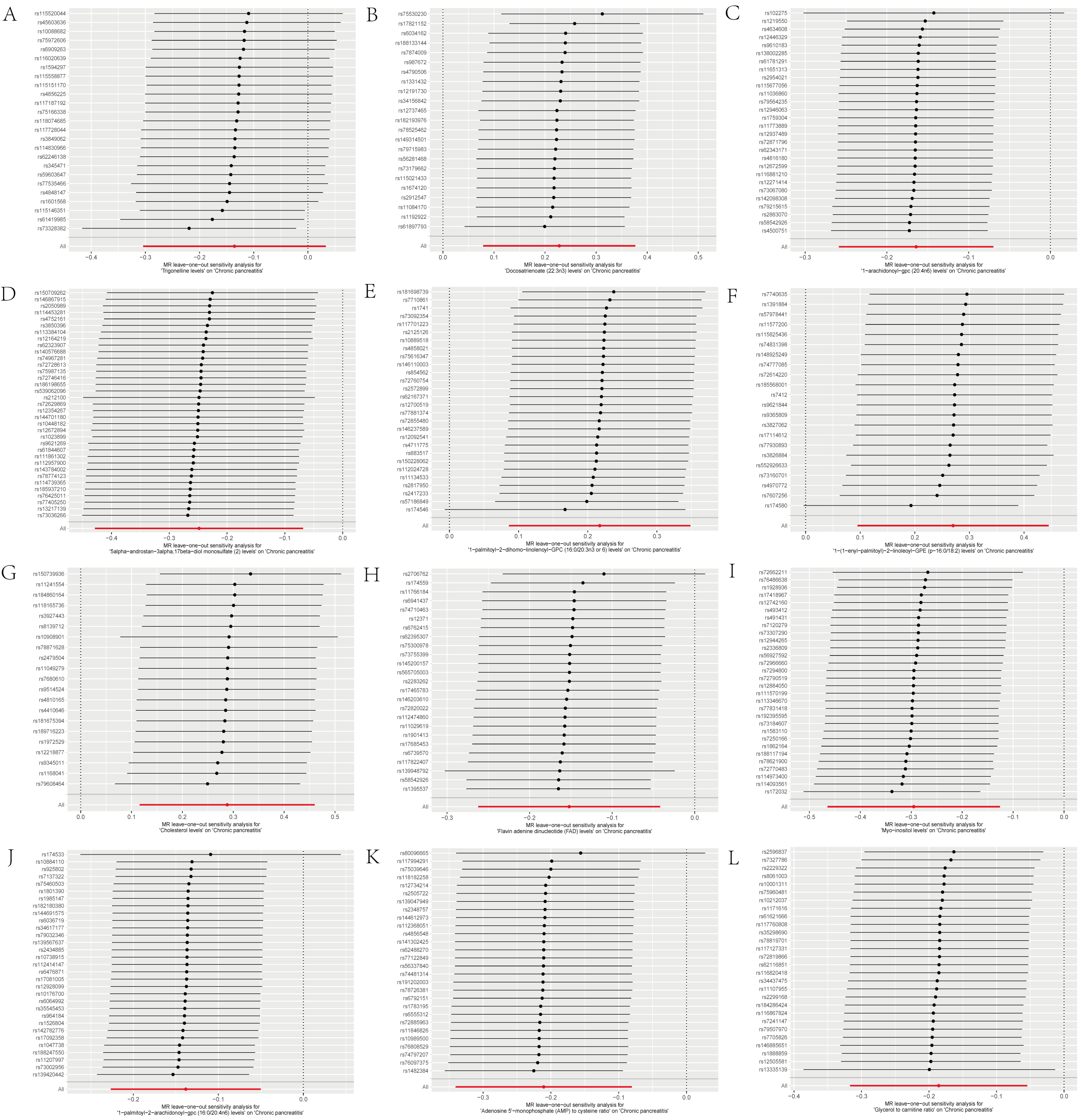

Supplement: Supplementary file 2 — Supplementary Material 2. [file 41065_2025_378_MOESM2_ESM.zip › Supplementary Figure 7.tif]

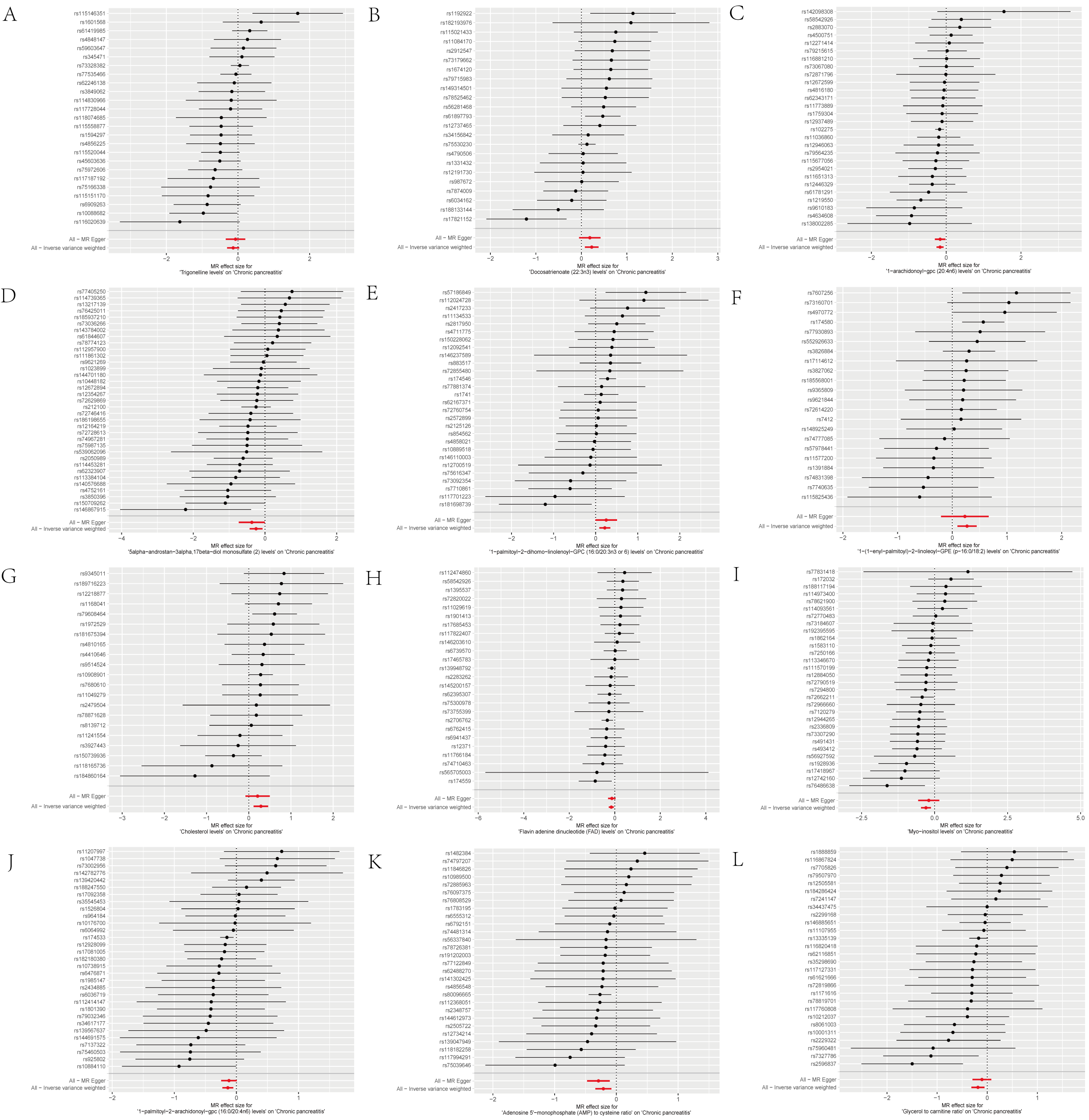

Supplement: Supplementary file 2 — Supplementary Material 2. [file 41065_2025_378_MOESM2_ESM.zip › Supplementary Figure 8.tif]
